# Prognostic significance of long non-coding RNAs in clear cell renal cell carcinoma: A meta-analysis

**DOI:** 10.1097/MD.0000000000017276

**Published:** 2019-10-04

**Authors:** Yan Wang, Zhan Li, Wei Li, Le Zhou, Yuehua Jiang

**Affiliations:** a Department of Nephrology, Affiliated Hospital of Shandong Academy of Medical Sciences, Shandong First Medical University; b Department of Cardiology, Shandong Provincial Qianfoshan Hospital, the First Hospital Affiliated with Shandong First Medical University; c Department of Nephrology, Affiliated Hospital of Shandong University of Traditional Chinese Medicine; d Central Laboratory, Affiliated Hospital of Shandong University of Traditional Chinese Medicine, Jinan, PR China.

**Keywords:** biomarker, clear cell renal cell carcinoma, long non-coding RNA, meta-analysis, prognosis

## Abstract

**Background:**

Clear cell renal cell carcinoma (ccRCC) is the most common form of kidney cancer in adults, and patients with advanced ccRCC have a 5-year survival rate of <30%. The poor prognosis of ccRCC is closely related to its lacking of potential therapeutic and prognostic biomarkers. This meta-analysis aimed to elucidate the precise prognostic value of long non-coding RNAs (lncRNAs) in patients with ccRCC.

**Methods:**

A literature search was performed in related databases up to January 31, 2019. Hazard ratios (HRs) and corresponding 95% confidence intervals (CIs) were calculated to explore the relationship between special lncRNAs expression and survival in patients with ccRCC.

**Results:**

After literature researching, a total of 16 studies, including 13 lncRNAs were identified. The data from studies that investigated the association between lncRNA expression and survival outcomes in patients with ccRCC were extracted. Results revealed that lncRNAs expression was significantly associated with poor overall survival (OS) outcome in patients with ccRCC (HR = 1.71, 95%CI = 1.40–2.01 in up-regulated subgroup; HR = 0.53, 95% CI = 0.25–0.80 in down-regulated subgroup). The overexpression of PVT1 was significantly associated with poor OS in ccRCC (HR = 1.51, 95% CI = 1.02–2.00). Meanwhile, up-regulation of LUCAT1 was significantly related to worse OS in ccRCC patients (HR = 1.51, 95% CI = 1.01–2.00).

**Conclusions:**

These results suggest that lncRNAs could be used to predict unfavorable prognosis and function as potential prognostic biomarkers in ccRCC.

## 1 Introduction

Renal cell carcinoma (RCC) is one of the most common malignant tumors in urinary system, accounting for about 5% of adult malignant tumors.^[1]^ Clear cell renal cell carcinoma (ccRCC) is the commonest subtype of RCC, which makes up approximately 3-fourths of RCC.^[2]^ Due to lacking of early diagnostic markers and the strong resistance of ccRCC to radiotherapy and chemotherapy, the overall prognosis of patients with ccRCC is still poor.^[3]^ In order to improve the prognosis, new markers need to be found for early diagnosis and decision-making related to the selection of appropriate treatment.

Long non-coding RNA (lncRNA) is a newly discovered class of non-coding RNA with >200 nucleotides in length. Accumulating studies have shown that lncRNAs are involved in many physiological and pathological processes, such as cell growth, apoptosis, stem cell pluripotency and development, by acting as transcriptional, post-transcriptional or epigenetic regulators.^[4,5]^ Recently, mounting evidences reveal that lncRNAs play important roles in human diseases, especially in tumorigenesis and tumor prognosis, which may be potential biomarkers and therapeutic targets.^[6,7]^

Currently, the special expression profiles of lncRNAs have been observed in ccRCC and dysregulated lncRNAs regulate oncogenesis and metastasis-associated genes.^[8,9]^ Therefore, lncRNAs might be feasible as diagnostic biomarkers and prognostic factors. In order to explore the correlation between dysregulated lncRNAs and prognosis of patients with ccRCC, we conducted this quantitative meta-analysis.

## 2 Methods

### 2.1 Trial selection

All published controlled clinical trials involving the relationship between lncRNAs and clinical values in ccRCC patients were collected. The analyzed data were clinicopathology and prognosis of ccRCC patients during the follow-up. Studies without original data related to survival outcome were excluded. All analyses were performed based on previously published studies. Thus, no ethic approval and informed consent were required in this study.

### 2.2 Search strategy

A literature search was performed in Pubmed and Web of Science (up to January 31, 2019), EMBASE (up to 2018), and Cochrane Controlled Trials Register (up to 2018) to identify relevant clinical controlled trials in English, which was conducted with the terms “lncRNA” and “renal cancer”. The detail searching strategy in Pubmed was ((long non conding RNA) *OR* lncRNA) AND (cancer *OR* tumor *OR* carcinoma) AND (renal *OR* kidney *OR* (renal cancer) *OR* (renal cell cancer) *OR* (renal cell carcinoma)). The studies involving the prognosis of patients with ccRCC were adopted for further screening. And studies from the bibliographies of retrieved trials were also scanned.

The inclusion criteria were formulated as follows:

(1) English literature;
(2) follow-up completed in ccRCC patients after surgery;
(3) studies that detected the expression level of lncRNA in tissue and presented the clinicopathological features, such as age, tumor diameter, tumor stage and so on;
(4) studies that investigated the association between the expression level of lncRNA and survival outcome;
(5) studies that provided a Hazard ratio (HR) or relative risk (RR), 95% confidence interval (CI) and *P* value, and Kaplan–Meier curves or required data obtained by contacting corresponding authors.

This meta-analysis adopted the following criteria to exclude irrelevant studies:

(1) non-human trials;
(2) non-English literature;
(3) studies performed only in cellular level;
(4) ccRCC patients ≤ 40 cases;
(5) studies included other type RCC patients, for example papillary renal cell carcinoma and chromophobic renal cell carcinoma;
(6) studies without follow-up information;
(7) studies focusing on lncRNA genetic alterations, including methylation patterns or polymorphisms;
(8) the analysis of HRs based on multiple lncRNAs;
(9) the duplicate data from The Cancer Genome Atlas; and
(10) studies that could not obtained original and sufficient data for HR and 95% CI estimation.

### 2.3 Data extraction

Two investigators (Wang Yan and Li Zhan) were blinded to each other when reviewing the trials and independently selected studies using the following steps:

(1) examining titles and abstracts to remove obviously irrelevant reports;
(2) retrieving the full text of potentially relevant reports;
(3) examining full-text reports for compliance of studies with eligibility criteria;
(4) making final decisions on study inclusion.

Any discrepancies were resolved by consensus. The quality assessment of these included studies was performed in accordance with the Quality Assessment of Diagnostic Accuracy Studies-2 (QUADAS-2) criteria. If a consensus could not be reached, the senior author (Li Wei) made the final decision for trial eligibility and data extraction. For each study, the following information was recorded: first author's name, year of publication, country, number of patients, median or mean age of patients, sample size and type, detection method, cutoff definition, follow-up time, and HRs associated with dysregulated lncRNAs expression for over survival (OS), disease-free survival (DFS) or progression free survival (PFS), along with their 95% CIs and *P* values.

### 2.4 Statistical analysis

The HRs and 95% CI of survival outcome were calculated for each study. In lncRNAs up-regulated group, HR > 1 implied a worse survival. Conversely, an observed HR < 1 suggested poor survival for lncRNA down-regulated group. A pooled effect was calculated using a random effect model to take into account within-study and between-study variance. Heterogeneity was assessed with the *Q* statistic (the result of a statistical test based on the *Q* statistic (*P*)) and the *I*^2^ statistic. For the *Q* test, a *P* value less than .05 indicated significant heterogeneity; for the *I*^2^ statistics, an *I*^2^ value greater than 50% was considered significant heterogeneity. If *I*^2^ value less than 50%, a fixed effect model was performed; otherwise, a random effect model was applied for the meta-analysis. While statistically significant heterogeneity was found, the source would be explored by subgroup analysis and/or meta-regression.

The sensitivity analysis was performed to examine the consistency of the overall effect estimate respectively and publication bias was examined using funnel plot. All statistical procedures in this meta-analysis were performed using STATA 14.0 and Revman 5.2. The *P* < .05 was considered as statistically significant.

## 3 Results

### 3.1 Search result

**Figure 1:**
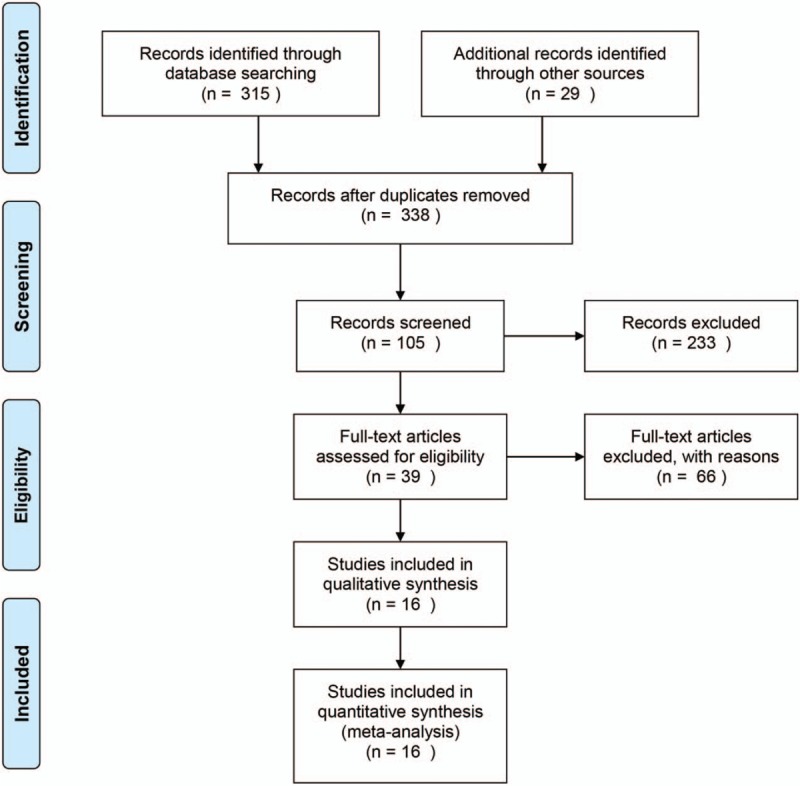
Flow diagram of study selection process.

As shown in Figure 1, 338 records relating to lncRNA expression and ccRCC were identified in PubMed, Embase, Web of Science, and Cochrane databases. After screening titles, 233 records were excluded. After the abstracts were reviewed, 39 full-text articles were assessed for eligibility. Finally, after excluding 23 articles, a total of 16 articles were included in the current meta-analysis.

### 3.2 Characteristics and clinicopathological features of eligible studies

The main features and results of the eligible articles were summarized in Tables 1 and 2. These studies investigated a total of 1290 cases. Among these 16 studies, 14 came from China, 1 from Japan, and 1 from Germany. Twelve studies only focused on OS, while other 4 studies calculated OS and DFS/PFS. A total of 13 lncRNAs were investigated in these 16 included studies. The lung cancer associated transcript 1 (LUCAT1),^[10,11]^ promoter of CDKN1A antisense DNA damage activated RNA (PANDAR),^[12]^ plasmacytoma variant translocation 1 (PVT1),^[13,14]^ metastatic renal cell carcinoma-associated transcript 1 (MRCCAT1),^[15]^ linc00152,^[16]^ lncRNA H19,^[17]^ metastasis-associated lung adenocarcinoma transcript 1 (MALAT1),^[18,19]^ protein sprouty homolog 4 intronic transcript-1 (SPRY4-IT1)^[20]^ were up-regulated. And ENSG241684,^[21]^ NONHSAT123350,^[22]^ lnc-ZNF180-2,^[23]^ neuroblastoma associated transcript-1 (NBAT1),^[24]^ and cell adhesion molecule 1 anti-sense transcript-1 (CADM1-AS1)^[25]^ were down-regulated. None of these studies clarified that these lncRNAs were related to gender and age of ccRCC patients. Zhang claimed that up-regulated MALAT1 were significantly correlated with tumor size.^[18]^ Two studies revealed that clinicopathological characteristics were not significantly associated with the expression levels of NONHSAT123350 and lnc-ZNF180-2.^[22,23]^ The other studies reported that lncRNAs were significantly associated with tumor stage (Table 1).

**Table 1:**
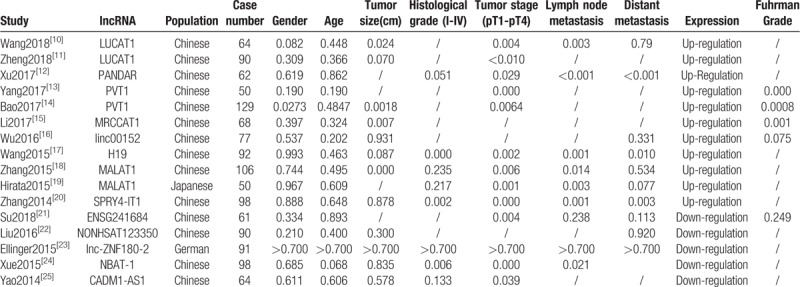
Summary of the comparison for the *P* values of the association between lncRNAs and clinicopathological features.

**Table 2:**
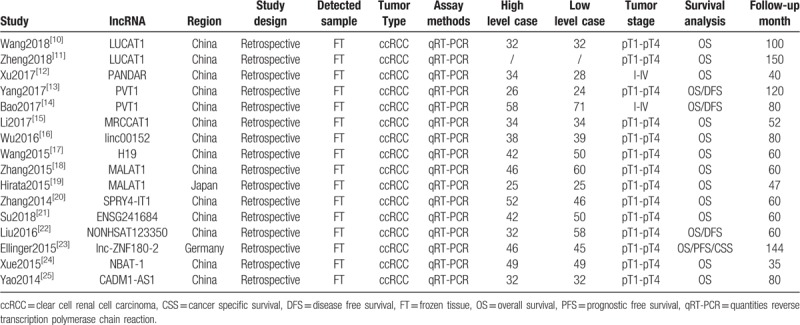
Summary of lncRNAs used as prognostic biomarkers of ccRCC.

### 3.3 Correlation between clinicopathological data and survival

The 16 included studies evaluated the lncRNA expression in 1290 tissue samples of ccRCC patients by quantitative real-time polymerase chain reaction (qRT-PCR). The clinicopathological characteristics of these 16 eligible studies were presented in Table 2. These 13 lncRNAs were all correlated with the prognosis of ccRCC patients. The overexpression of PANDAR, lncRNA H19, PVT1, linc00152, LUCAT1, MRCCAT1, MALAT1, and SPRY4-IT1 were associated with poor prognosis; meanwhile the low expressions of CADM1-AS1, ENSG241684, lnc-ZNF180-2, NONHSAT123350, NBAT1 were related to poor prognosis.

**Figure 2:**
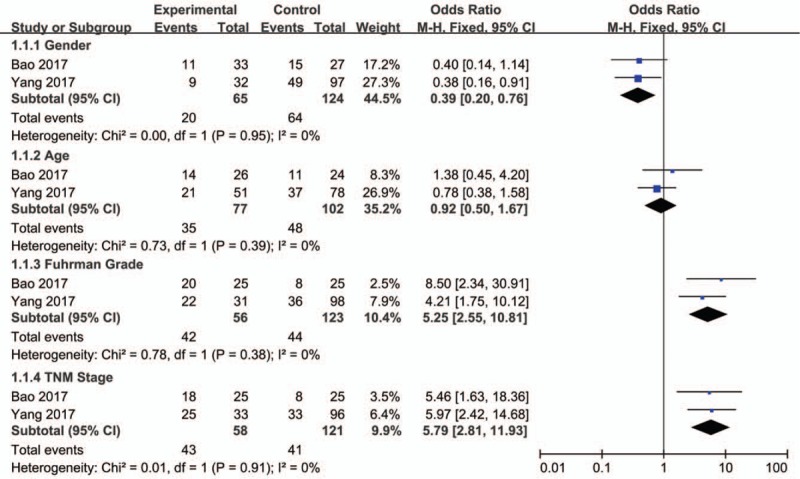
Forest plots of studies evaluating odds ratios (ORs) of PVT1 expression and the clinicopathology features of patients with ccRCC. ccRCC = clear cell renal cell carcinoma.

MALAT1, PVT1, and LUCAT1 were respectively detected in 2 studies. The characteristics of studies on MALAT1 have been performed.^[26]^ And the studies on LUCAT1 did not include the relationship between LUCAT1 expression and clinicopathological features in patients with ccRCC. We then combined the 2 studies on PVT1 with 4 groups to clarify the correlation between clinicopathological data and PVT1 level. After meta-analysis of these 2 studies, we found that up-regulated PVT1 was significantly associated with gender (HR = 0.39, 95% CI = 0.20–0.76, Fig. 2), Fuhrman grade (HR = 5.25, 95% CI = 2.55–10.81, Fig. 2), and TNM stage (HR = 5.79, 95% CI = 2.81–11.93, Fig. 2). However, the overexpression of PVT1 was not correlated with age (HR = 0.92, 95% CI = 0.50–1.67, Fig. 2).

### 3.4 Correlation between lncRNAs expression and prognosis

#### 3.4.1 Association between lncRNAs and OS

**Figure 3:**
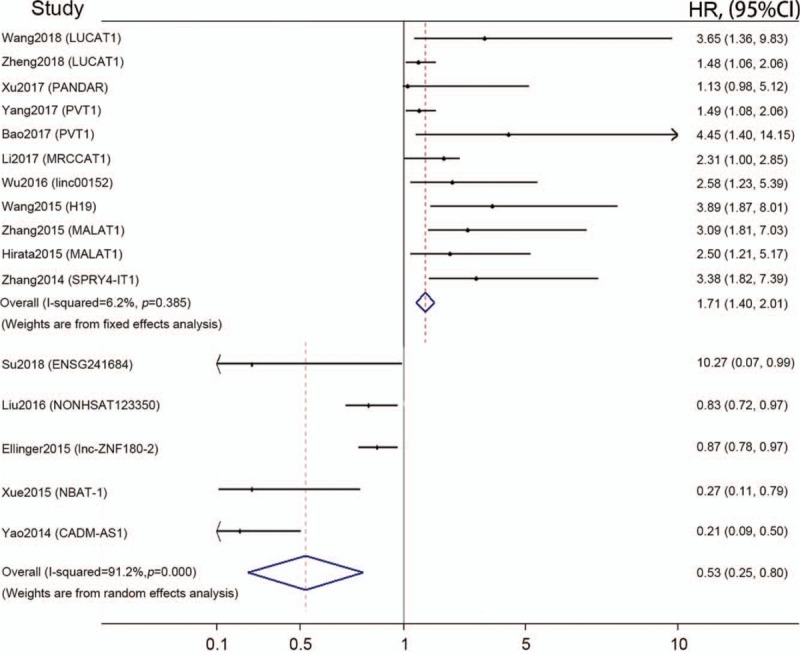
Forest plot summarizing the association between lncRNAs expression and overall survival in patients with ccRCC. ccRCC = clear cell renal cell carcinoma, CI = confidence interval, HR = Hazard ratio.

These 16 articles evaluating OS were divided into up-regulated subgroup and down-regulated subgroup according to the lncRNAs expression level in patients with ccRCC. Significant heterogeneity between included studies was observed in down-regulated subgroup data (*I*^2^ = 91.2%). Therefore, the random effect model was used in the meta-analysis of down-regulated subgroup to calculate the pooled HR and 95% CI. While the fixed effect model was used for up-regulated subgroup (*I*^2^ = 6.2%). The results showed that expression of lncRNAs was significantly associated with poor OS outcome in patients with ccRCC, with a pooled HR of 1.71 (95% CI = 1.40–2.01; Fig. 3) in up-regulated subgroup and a pooled HR of 0.53 (95% CI = 0.25–0.80; Fig. 3) in down-regulated subgroup.

**Figure 4:**
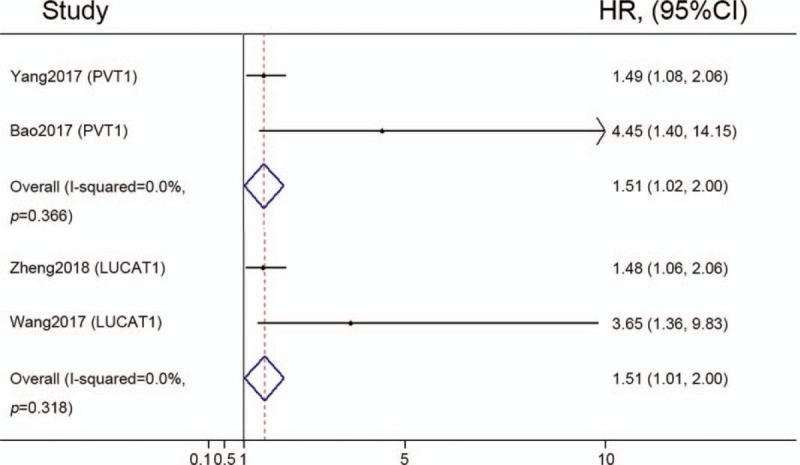
Forest plots representing subgroup analyses of the relationship between overall survival and PVT1/LUCAT1 expression in patients with ccRCC. ccRCC = clear cell renal cell carcinoma, CI = confidence interval, HR = Hazard ratio.

The association between the expression level of MALAT1 and OS has been performed.^[26]^ We then combined the two studies separately on PVT1 and LUCAT1 to clarify the correlation between the expression level of lncRNAs and OS. As heterogeneity was not apparent in the OS analysis of PVT1 group (*I*^2^ = 0.0%, *P* = .366) and LUCAT1 group (*I*^2^ = 0.0%, *P* = .318), we used a fixed effect model to pool HRs. After meta-analysis of these studies, we found that up-regulation of PVT1 was significantly associated with poor OS in patients with ccRCC (HR = 1.51, 95% CI = 1.02–2.00, Fig. 4). Meanwhile, up-regulation of LUCAT1 was significantly related to worse OS in ccRCC (HR = 1.51, 95% CI = 1.01–2.00, Fig. 4).

#### 3.4.2 Association between lncRNAs and DFS/PFS

**Figure 5:**
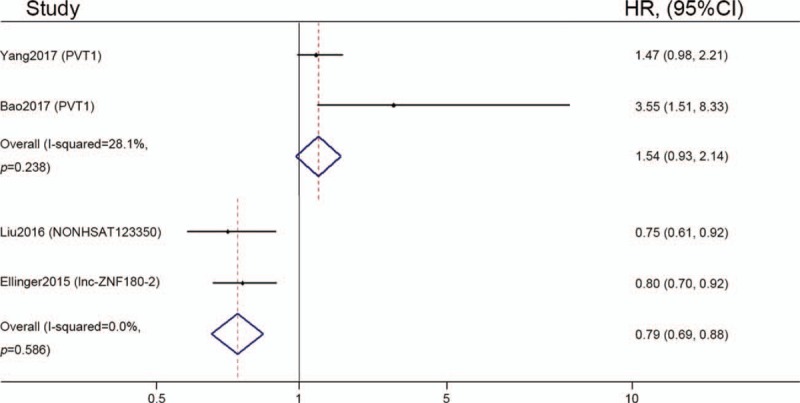
Forest plot of studies having assessed the association between lncRNAs expression and disease-/progression-free survival of patients with ccRCC. ccRCC = clear cell renal cell carcinoma, CI = confidence interval, HR = Hazard ratio.

Among the 4 articles evaluating DFS/PFS, no statistically significant heterogeneity was evident (*I*^2^ = 28.1%, *P* = .238 in up-regulated subgroup; *I*^2^ = 0.0%, *P* = .586 in down-regulated subgroup). Therefore, a fixed effect model was used to calculate the pooled HR and 95% CI. The overexpression of PVT1 was found to be not significantly correlated with poor DFS /PFS in patients with ccRCC, with a pooled HR of 1.54 (95% CI = 0.93–2.14; Fig. 5). Moreover, the low expressions of NONHSAT123350 and lnc-ZNF180-2 were significantly associated with poor DFS/PFS outcome in patients with ccRCC, with a pooled HR of 0.79 (95% CI = 0.69–0.88; Fig. 5).

### 3.5 Heterogeneity analysis and publication bias

**Figure 6:**
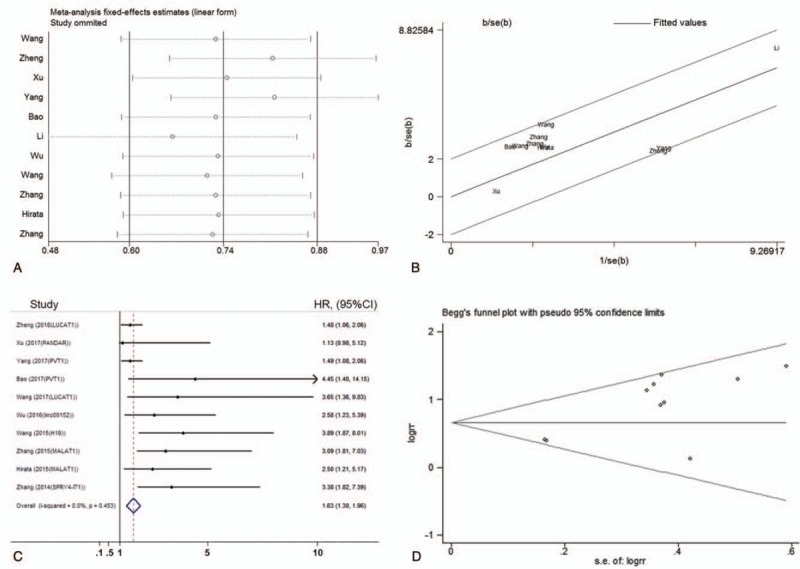
Sensitivity analysis, Galbraith radial plots and Begg test for heterogeneity conducted in up-regulated subgroup. (A) Sensitivity analysis of studies measured OS in up-regulated subgroup; (B) Galbraith radial plot of studies measured OS in up-regulated subgroup; (C) Forest plot of studies evaluated the association between lncRNAs expression and OS of patients with ccRCC, following exclusion of data from Li2017; (D) Begg's funnel plot relating to analysis of OS in up-regulated subgroup to assess publication bias. ccRCC = clear cell renal cell carcinoma, CI = confidence interval, DFS = disease-free survival, HR = Hazard ratio, OS = overall survival, PFS = progression-free survival.

Sensitivity analysis was performed by meta-based Influence Analysis using the fixed effect model in OS analysis of up-regulated subgroup. This test suggested that the Li2017 study had influenced the overall result of the OS analysis (Fig. 6A). When the Galbraith plot was assessed, Li2017 study was identified as an outlier causing heterogeneity in the OS analysis (Fig. 6B). By excluding this study, a similar and significant pooled HR was obtained (HR = 1.63, 95% CI = 1.30–1.96). Moreover, heterogeneity was found to be absent (*I*^2^ = 0.0%, *P* = .453; Fig. 6C). Begg's funnel plots were used to evaluate publication bias (Fig. 6D). For the pooled analyses of OS in up-regulated subgroup, the Begg test showed a *P* value of .283 after continuity corrected. Therefore, this meta-analysis was free of notable publication bias. No conclusive graph could be generated because of the small size of down-regulated subgroup and DFS/PFS analysis. So, the heterogeneity and publication bias analyses were not evaluated.

## 4 Discussion

Non-coding RNAs profiling has been found to be useful in the prediction of cancer patient's clinical outcome, metastatic recurrence and survival time.^[27,28]^ During carcinogenesis, genetic alterations could drive tumor evolution toward higher grades of malignancy, however, the extent to how the lncRNAs alterations influence this process remains incompletely understood. The up-regulation or down-regulation of many lncRNAs, such as MALAT1 and H19, contributed to oncogenesis by affecting proliferation, energetic metabolism and other cellular processes.^[29–31]^ Many long non-coding RNAs have been identified in various cancer genomes, and can be used as novel biomarkers for cancer.^[32]^ As multiple reports suggest, lncRNAs play an important role in urothelial carcinogenesis, such as bladder cancer.^[33]^ Some lncRNAs, for example, MAGI2-AS3 and ADAMTS9-AS2 may serve as candidate diagnostic biomarkers or therapeutic targets for bladder cancer.^[34]^

The pathogenesis and progression of renal cancer involve multistep changes in the gene profiling, and a variety of approaches have been used to uncover the molecular profiles that contribute to ccRCC development and tumor progression. Many metabolic pathways in ccRCC are reprogrammed.^[35]^ The metabolomic profile demonstrated that mitochondrial dysfunction and oxidative stress potentially associated with tumorigenesis and tumor progression.^[36]^ The specific molecular alterations occur in ccRCC under metabolic reprogramming, but the role of lncRNAs is still unclear.^[37]^ The potential value of lncRNAs for ccRCC remains further investigation. In order to find some biomarkers for ccRCC, we conducted this meta-analysis to systematically analyze the association between lncRNA expression and prognosis of patients with ccRCC.

After literature searching and screening, a total of 16 studies were included. In the classification of clinicopathological features, most lncRNAs have little association with the tumor size. The up-regulated PANDAR, H19, SPRY4-IT1 could be potentially considered as novel biomarkers to detect distant metastasis in ccRCC patients in early stages. The dysregulated PANDAR, NBAT-1, H19, LUCAT1, lnc-ZNF180-2, MALAT1, SPRY4-IT1 might be used as biomarkers of lymph node metastasis. Except MALAT1, PVT1, and LUCAT1, the other lncRNAs were detected by single study. The characteristics of studies on MALAT1 have been performed.^[26]^ And the studies on LUCAT1 did not included the relationship between LUCAT1 expression and clinicopathological features in patients with ccRCC. We then combined the 2 studies on PVT1 with 4 groups to clarify the correlation between clinicopathological data and PVT1 level. The expression of this lncRNA was related to the gender of patients with ccRCC but not age. PVT1 was significantly associated with the Fuhrman grade and TNM stage of patients with ccRCC after we pooled HR and *P* value. Because of the limitations of included studies, further studies should be conducted to verify these conclusions.

Among these 14 lncRNAs, the overexpression of PANDAR, lncRNA H19, PVT1, linc00152, LUCAT1, MRCCAT1, MALAT1, SPRY4-IT1, and lncRNA MIAT were associated with poor prognosis; meanwhile the low expression of CADM1-AS1, ENSG241684, lnc-ZNF180-2, NONHSAT123350, NBAT1 were related to poor prognosis. Although these dysregulated lncRNAs were associated with the prognosis of patients with ccRCC, merely MALAT1, PVT1, and LUCAT1 were not reported solely. The association between the expression level of MALAT1 and OS has been performed.^[26]^ We then conducted meta-analyses to elucidate the relationship between expression level of the other 2 lncRNAs and the OS of patients with ccRCC. The results revealed that overexpression of PVT1 predicted poor survival among patients with ccRCC; as well as patients with up-regulated LUCAT1 exhibited short OS. However, due to the limited size of related studies, further research should be performed to confirm this conclusion.

In this study, PVT1 is among those lncRNAs, which is overexpressed and associated with tumorigenesis and poor prognosis in a range of cancers.^[38,39]^ Chen et al found that overexpression of PVT1 could promote the proliferation, cell cycle progression, and migration of melanoma cells.^[40]^ Moreover, Gao et al clarified that after transfected with PVT1 siRNA, the proliferation, migration, and invasion of cervical cancer cells were greatly decreased.^[41]^ The overexpressed PVT1 regulated miR-195 pathway to influence lipid metabolism and the metabolic reprogramming was verified to be potential mechanism to promote proliferation and invasion of tumor cells.^[42]^ The increased expression of PVT1 was correlated with advanced TNM stage, histological grade, and poor survival of ccRCC. Wu et al showed that PVT1 inhibited renal cancer cell apoptosis by enhancing the stability of Myeloid cell leukemia-1 mRNA.^[43]^ Li et al revealed that the expression of PVT1 was up-regulated in ccRCC tissues, and knockdown of PVT1 induced apoptosis by increasing the expression of poly ADP ribose polymerase and Bcl-2-associated X protein, and promoted cell cycle arrest at the G1 phase by decreasing the expression of cyclin D1. This study indicated that PVT1 promoted the progression of ccRCC partly through activation of the epidermal growth factor receptor pathway.^[44]^ These findings suggest that PVT1 may be an oncogenic biomarker for ccRCC, and the PVT1 associated pathway may serve as a novel therapeutic target for treating ccRCC.

Our study revealed that LUCAT1 was correlated not only with clinicopathological features but also with prognosis of ccRCC. Xiao et al revealed that the up-regulated expression of LUCAT1 in ccRCC tissues was associated with tumor grade, clinical pathological stage and survival time; LUCAT1 could bind to polycomb PRC2 complex and suppress p57 expression to promote renal cancer cell proliferation.^[45]^ Zheng et al clarified that the expression level of LUCAT1 was positively correlated with malignant stage and poor prognosis of ccRCC, and LUCAT1 promoted proliferation and invasion in ccRCC cells through AKT/GSK-3β signaling pathway.^[11]^ Wang found that LUCAT1 was critical for proliferation and invasion of ccRCC cells by regulating micoRNA-495-3p and special adenine-thymine-rich DNA-binding protein 1.^[10]^ These findings indicate that the LUCAT1 related axis is a potential therapeutic target and molecular biomarker for ccRCC.

This meta-analysis summarized current researches on the relationship between aberrant lncRNAs expression and prognosis of patients with ccRCC. However, several limitations should be considered. First, the number of studies included in our meta-analysis was insufficient and the sample size was limited. Second, most included studies were from China, merely one from Japan, and one from Germany; therefore, our data might not be globally applicable. Thirdly, the standard for dysregulated expression differed among these studies, and it was difficult to obtain the same value. Consequently, further studies and more rigorous criteria will be needed to confirm the function of lncRNAs in ccRCC.

## 5 Conclusion

In conclusion, our meta-analyses indicate that the expression levels of some special lncRNAs are correlated with poor survival in patients with ccRCC. The expression of lncRNAs could be used to predict unfavorable prognosis and function as potential prognostic biomarkers in ccRCC.

## Author contributions

**Data curation:** Yan Wang, Zhan Li.

**Methodology:** Yan Wang, Zhan Li, Le Zhou, Yuehua Jiang.

**Supervision:** Wei Li.

**Writing – original draft:** Yan Wang.

**Writing – review & editing:** Zhan Li, Wei Li.

## References

[1] Siegel R Ma J Zou Z Cancer statistics, 2014. CA Cancer J Clin 2014;64:9–29. 24399786 10.3322/caac.21208

[2] Escudier B Porta C Schmidinger M Renal cell carcinoma: ESMO Clinical Practice Guidelines for diagnosis, treatment and follow-up. Ann Oncol 2016;27:v58–68. 27664262 10.1093/annonc/mdw328

[3] Motzer RJ Escudier B McDermott DF Nivolumab versus everolimus in advanced renal-cell carcinoma. N Engl J Med 2015;373:1803–13. 26406148 10.1056/NEJMoa1510665 PMC5719487

[4] Gibb EA Brown CJ Lam WL The functional role of long non-coding RNA in human carcinomas. Mol Cancer 2011;10:38. 21489289 10.1186/1476-4598-10-38 PMC3098824

[5] Li X Wu Z Fu X Long noncoding RNAs: insights from biological features and functions to diseases. Med Res Rev 2013;33:517–53. 22318902 10.1002/med.21254

[6] Wang A Bao Y Wu Z Long noncoding RNA EGFR-AS1 promotes cell growth and metastasis via affecting HuR mediated mRNA stability of EGFR in renal cancer. Cell Death Dis 2019;10:154. 30770799 10.1038/s41419-019-1331-9 PMC6377662

[7] Prensner JR Chinnaiyan AM The emergence of lncRNAs in cancer biology. Cancer Discov 2011;1:391–407. 22096659 10.1158/2159-8290.CD-11-0209 PMC3215093

[8] Dong D Mu Z Wei N Long non-coding RNA ZFAS1 promotes proliferation and metastasis of clear cell renal cell carcinoma via targeting miR-10a/SKA1 pathway. Biomed Pharmacother 2019;111:917–25. 30841471 10.1016/j.biopha.2018.12.143

[9] Yang S Xu J Zeng X A six-long non-coding RNA signature predicts prognosis in melanoma patients. Int J Oncol 2018;52:1178–88. 29436619 10.3892/ijo.2018.4268 PMC5843393

[10] Wang LN Zhu XQ Song XS Long noncoding RNA lung cancer associated transcript 1 promotes proliferation and invasion of clear cell renal cell carcinoma cells by negatively regulating miR-495-3p. J Cell Biochem 2018;119:7599–609. 29932248 10.1002/jcb.27099

[11] Zheng Z Zhao F Zhu D Long non-coding RNA LUCAT1 promotes proliferation and invasion in clear cell renal cell carcinoma through AKT/GSK-3beta signaling pathway. Cell Physiol Biochem 2018;48:891–904. 30032137 10.1159/000491957

[12] Xu Y Tong Y Zhu J An increase in long non-coding RNA PANDAR is associated with poor prognosis in clear cell renal cell carcinoma. BMC Cancer 2017;17:373. 28545465 10.1186/s12885-017-3339-9 PMC5445460

[13] Yang T Zhou H Liu P lncRNA PVT1 and its splicing variant function as competing endogenous RNA to regulate clear cell renal cell carcinoma progression. Oncotarget 2017;8:85353–67. 29156724 10.18632/oncotarget.19743 PMC5689614

[14] Bao X Duan J Yan Y Upregulation of long noncoding RNA PVT1 predicts unfavorable prognosis in patients with clear cell renal cell carcinoma. Cancer Biomark 2017;21:55–63. 29081406 10.3233/CBM-170251 PMC13075746

[15] Li JK Chen C Liu JY Long noncoding RNA MRCCAT1 promotes metastasis of clear cell renal cell carcinoma via inhibiting NPR3 and activating p38-MAPK signaling. Mol Cancer 2017;16:111. 28659173 10.1186/s12943-017-0681-0 PMC5490088

[16] Wu Y Tan C Weng WW Long non-coding RNA Linc00152 is a positive prognostic factor for and demonstrates malignant biological behavior in clear cell renal cell carcinoma. Am J Cancer Res 2016;6:285–99. 27186403 PMC4859660

[17] Wang L Cai Y Zhao X Down-regulated long non-coding RNA H19 inhibits carcinogenesis of renal cell carcinoma. Neoplasma 2015;62:412–8. 25866221 10.4149/neo_2015_049

[18] Zhang HM Yang FQ Chen SJ Upregulation of long non-coding RNA MALAT1 correlates with tumor progression and poor prognosis in clear cell renal cell carcinoma. Tumour Biol 2015;36:2947–55. 25480417 10.1007/s13277-014-2925-6

[19] Hirata H Hinoda Y Shahryari V Long noncoding RNA MALAT1 promotes aggressive renal cell carcinoma through Ezh2 and interacts with miR-205. Cancer Res 2015;75:1322–31. 25600645 10.1158/0008-5472.CAN-14-2931 PMC5884967

[20] Zhang HM Yang FQ Yan Y High expression of long non-coding RNA SPRY4-IT1 predicts poor prognosis of clear cell renal cell carcinoma. Int J Clin Exp Pathol 2014;7:5801–9. 25337221 PMC4203192

[21] Su H Wang H Shi G Downregulation of long non-coding RNA ENSG00000241684 is associated with poor prognosis in advanced clear cell renal cell carcinoma. Eur J Surg Oncol 2018;44:840–6. 29433989 10.1016/j.ejso.2018.01.013

[22] Liu H Chen P Jiang C Screening for the key lncRNA targets associated with metastasis of renal clear cell carcinoma. Medicine (Baltimore) 2016;95:e2507. 26765468 10.1097/MD.0000000000002507 PMC4718294

[23] Ellinger J Alam J Rothenburg J The long non-coding RNA lnc-ZNF180-2 is a prognostic biomarker in patients with clear cell renal cell carcinoma. Am J Cancer Res 2015;5:2799–807. 26609485 PMC4633906

[24] Xue S Li QW Che JP Decreased expression of long non-coding RNA NBAT-1 is associated with poor prognosis in patients with clear cell renal cell carcinoma. Int J Clin Exp Pathol 2015;8:3765–74. 26097558 PMC4466945

[25] Yao J Chen Y Wang Y Decreased expression of a novel lncRNA CADM1-AS1 is associated with poor prognosis in patients with clear cell renal cell carcinomas. Int J Clin Exp Pathol 2014;7:2758–67. 25031695 PMC4097296

[26] Chen J Chen Y Gu L LncRNAs act as prognostic and diagnostic biomarkers in renal cell carcinoma: a systematic review and meta-analysis. Oncotarget 2016;7:74325–36. 27527868 10.18632/oncotarget.11101 PMC5342056

[27] van’t Veer LJ Bernards R Enabling personalized cancer medicine through analysis of gene-expression patterns. Nature 2008;452:564–70. 18385730 10.1038/nature06915

[28] Peng F Wang R Zhang Y Differential expression analysis at the individual level reveals a lncRNA prognostic signature for lung adenocarcinoma. Mol Cancer 2017;16:98. 28587642 10.1186/s12943-017-0666-z PMC5461634

[29] Ma L Bajic VB Zhang Z On the classification of long non-coding RNAs. RNA Biol 2013;10:925–33. 23696037 10.4161/rna.24604 PMC4111732

[30] Ji E Kim C Kim W Role of long non-coding RNAs in metabolic control. Biochim Biophys Acta Gene Regul Mech 2018;pii:S1874-9399(18)30460-7. Epub ahead of print. 10.1016/j.bbagrm.2018.12.006 30594638

[31] Sun H Huang Z Sheng W Emerging roles of long non-coding RNAs in tumor metabolism. J Hematol Oncol 2018;11:106. 30134946 10.1186/s13045-018-0648-7 PMC6104013

[32] Lavorgna G Vago R Sarmini M Long non-coding RNAs as novel therapeutic targets in cancer. Pharmacol Res 2016;110:131–8. 27210721 10.1016/j.phrs.2016.05.018

[33] Terracciano D Ferro M Terreri S Urinary long noncoding RNAs in nonmuscle-invasive bladder cancer: new architects in cancer prognostic biomarkers. Transl Res 2017;184:108–17. 28438520 10.1016/j.trsl.2017.03.005

[34] Zhu N Hou J Wu Y Integrated analysis of a competing endogenous RNA network reveals key lncRNAs as potential prognostic biomarkers for human bladder cancer. Medicine (Baltimore) 2018;97:e11887. 30170380 10.1097/MD.0000000000011887 PMC6392549

[35] Lucarelli G Loizzo D Franzin R Metabolomic insights into pathophysiological mechanisms and biomarker discovery in clear cell renal cell carcinoma. Expert Rev Mol Diagn 2019;19:397–407. 30983433 10.1080/14737159.2019.1607729

[36] Lucarelli G Rutigliano M Sallustio F Integrated multi-omics characterization reveals a distinctive metabolic signature and the role of NDUFA4L2 in promoting angiogenesis, chemoresistance, and mitochondrial dysfunction in clear cell renal cell carcinoma. Aging (Albany NY) 2018;10:3957–85. 30538212 10.18632/aging.101685 PMC6326659

[37] Soltysova A Breza J Takacova M Deregulation of energetic metabolism in the clear cell renal cell carcinoma: a multiple pathway analysis based on microarray profiling. Int J Oncol 2015;47:287–95. 25998032 10.3892/ijo.2015.3014

[38] Kong R Zhang EB Yin DD Long noncoding RNA PVT1 indicates a poor prognosis of gastric cancer and promotes cell proliferation through epigenetically regulating p15 and p16. Mol Cancer 2015;14:82. 25890171 10.1186/s12943-015-0355-8 PMC4399399

[39] Cui D Yu CH Liu M Long non-coding RNA PVT1 as a novel biomarker for diagnosis and prognosis of non-small cell lung cancer. Tumour Biol 2016;37:4127–34. 26490983 10.1007/s13277-015-4261-x

[40] Chen X Gao G Liu S Long noncoding RNA PVT1 as a novel diagnostic biomarker and therapeutic target for melanoma. Biomed Res Int 2017;2017:7038579. 28265576 10.1155/2017/7038579 PMC5318621

[41] Gao YL Zhao ZS Zhang MY Long noncoding RNA PVT1 facilitates cervical cancer progression via negative regulating of miR-424. Oncol Res 2017;25:1391–8. 28276314 10.3727/096504017X14881559833562 PMC7841064

[42] Song J Wu X Liu F Long non-coding RNA PVT1 promotes glycolysis and tumor progression by regulating miR-497/HK2 axis in osteosarcoma. Biochem Biophys Res Commun 2017;490:217–24. 28602700 10.1016/j.bbrc.2017.06.024

[43] Wu Q Yang F Yang Z Long noncoding RNA PVT1 inhibits renal cancer cell apoptosis by up-regulating Mcl-1. Oncotarget 2017;8:101865–75. 29254209 10.18632/oncotarget.21706 PMC5731919

[44] Li W Zheng Z Chen H Knockdown of long non-coding RNA PVT1 induces apoptosis and cell cycle arrest in clear cell renal cell carcinoma through the epidermal growth factor receptor pathway. Oncol Lett 2018;15:7855–63. 29725475 10.3892/ol.2018.8315 PMC5920359

[45] Xiao H Bao L Xiao W Long non-coding RNA Lucat1 is a poor prognostic factor and demonstrates malignant biological behavior in clear cell renal cell carcinoma. Oncotarget 2017;8:113622–34. 29371934 10.18632/oncotarget.21185 PMC5768351

